# Severe Alcohol-Related Liver Disease With Features Suggestive of Cirrhosis in a Young Adult Following Short-Term High-Risk Alcohol Use: A Case Report

**DOI:** 10.7759/cureus.97601

**Published:** 2025-11-23

**Authors:** Winluck Shayo, Min Kyaw Han, Rimsha Ghufran, Zaw Thant Lwin

**Affiliations:** 1 Acute Medicine, University Hospitals of Derby and Burton NHS Foundation Trust, Derby, GBR; 2 Internal Medicine, University Hospitals of Derby and Burton NHS Foundation Trust, Derby, GBR

**Keywords:** alcohol misuse, alcohol-related liver disease, binge drinking, cirrhosis, early-onset arld, hepatology, liver fibrosis, psychosocial stressors, withdrawal management, young adult

## Abstract

Alcohol-related liver disease (ARLD) typically develops after many years of harmful alcohol use, and features suggestive of cirrhosis in individuals under 30 are considered uncommon.

We describe a 24-year-old man admitted with acute alcohol withdrawal. He reported an escalation of alcohol intake after the loss of three family members within an eight-month period, consuming approximately 119 units (≈ 952 grams of alcohol) per week, mainly from miniature vodka bottles and daily cocktails (13% ABV). Prior to this period, he drank socially without known liver disease.

On examination, he was alert and oriented with no evidence of hepatic encephalopathy. He had hand tremors and jaundice, with a palpable, firm liver edge suggestive of chronic liver disease. Laboratory tests showed: bilirubin 170 µmol/L (normal <21), alanine aminotransferase (AST) 182 U/L (normal <40), alkaline phosphatase (ALP) 375 U/L, gamma-glutamyl transferase (γ-GT) 4,464 U/L, albumin 34 g/L, international normalised ratio (INR) 1.19, hemoglobin 129 g/L, and platelets 93 ×10⁹/L. Ultrasound demonstrated features suggestive of cirrhosis, including a coarse, nodular liver surface and splenomegaly.

He received a standard alcohol withdrawal management regimen and psychosocial support, including bereavement counseling and referral to community alcohol services. He was discharged after seven days with ongoing hepatology follow-up, including outpatient fibrosis assessment.

This case illustrates severe ARLD with features suggestive of early cirrhosis in a very young adult after a relatively short period of high-risk alcohol consumption. While cirrhosis risk is typically linked to cumulative lifetime alcohol exposure, recent reports suggest a rising incidence of early-onset ARLD in individuals under 40, potentially influenced by genetic predisposition, binge-drinking patterns, and psychosocial stressors.

Clinicians should be aware that clinically significant liver injury may occur rapidly in young adults with high-risk drinking patterns, and that features suggestive of cirrhosis can develop earlier than traditionally expected. Early recognition, intervention, and psychosocial support remain essential to reduce the growing burden of ARLD among younger populations.

## Introduction

Alcohol-related liver disease (ARLD) is a major global health concern, contributing significantly to liver-related morbidity and mortality. It encompasses a spectrum of hepatic injury ranging from steatosis and alcoholic hepatitis to advanced fibrosis and hepatocellular carcinoma. Cirrhosis, the end stage of chronic liver injury, is characterized by irreversible fibrosis, regenerative nodules, and progressive loss of hepatic function. Traditionally, cirrhosis is considered a disease of middle-aged or older adults, developing after long periods of sustained alcohol misuse. However, recent epidemiological trends suggest a concerning rise in early-onset ARLD among individuals under 40, with reports describing liver disease with features suggestive of cirrhosis in patients under 30 years of age [1,2].

The pathophysiology of advanced alcohol-related liver disease involves repeated hepatic injury that triggers chronic inflammation, activation of hepatic stellate cells, and deposition of extracellular matrix. This leads to architectural distortion, portal hypertension, and impaired liver function. Although hepatocytes possess regenerative capacity, sustained injury surpasses this threshold, resulting in progressive fibrosis and, in some cases, cirrhosis. Portal hypertension, synthetic dysfunction, and immune dysregulation are hallmark features of decompensated cirrhosis [3,4].

In the UK, liver disease mortality has increased fourfold over the past 50 years, with alcohol-related liver disease being a major contributor [5]. Data from the Office for Health Improvement & Disparities show that mortality from alcoholic liver disease in individuals under 75 continues to rise, particularly in socioeconomically deprived areas [6]. Globally, ARLD accounts for approximately 3 million deaths annually, representing nearly 6% of all deaths [2].

This case is particularly notable due to the development of severe alcohol-related liver disease with features suggestive of early cirrhosis in a previously healthy 24-year-old man, following less than one year of high-volume alcohol consumption. While most cases of advanced liver disease in young adults are associated with congenital or autoimmune etiologies, this case illustrates the potential for rapid hepatic decompensation influenced by binge drinking and psychosocial stressors. Similar cases have been reported in addiction medicine settings, where features consistent with cirrhosis were described in patients under 35 with fewer than 10 years of alcohol misuse [7].

## Case presentation

A 24-year-old man was admitted to the hospital with acute alcohol withdrawal, presenting with tremors, agitation, and insomnia. He reported a dramatic escalation in alcohol intake over the preceding eight months, following the loss of three close family members. His consumption reached approximately 119 units per week (≈ 952 grams of alcohol), primarily from miniature vodka bottles and daily cocktails (13% ABV). Before this period, he had been drinking only socially for four years and had no known prior liver disease or other medical conditions.

On examination, he was alert and oriented, exhibiting hand tremors and scleral icterus, with no evidence of hepatic encephalopathy. Abdominal examination revealed a palpable, firm liver edge without ascites or clinically apparent splenomegaly. There were no stigmata of chronic liver disease, such as spider nevi or palmar erythema. Neurological examination was normal, and there were no signs of hepatic encephalopathy.

He was hemodynamically stable and afebrile. A Clinical Institute Withdrawal Assessment for Alcohol (CIWA-Ar) score of 18 indicated moderate alcohol withdrawal severity. He had a Maddrey Discriminant Function score of 27, which is associated with a favorable short-term prognosis. He was admitted for inpatient alcohol withdrawal management and further evaluation of liver function.

Investigations

Initial laboratory investigations (Table 1) revealed significant hepatic dysfunction: bilirubin 170 µmol/L (normal <21), the aspartate aminotransferase (AST) 270 U/L which was almost double the alanine aminotransferase (ALT) 182 U/L (normal <40), alkaline phosphatase 375 U/L, γ-glutamyl transferase (γ-GT) 4464 U/L, albumin 34 g/L, international normalised ratio (INR) 1.19, haemoglobin 129 g/L, and platelets 93 ×10⁹/L. These findings suggested marked hepatocellular injury, cholestasis, and early synthetic impairment. 

**Table 1:** Serial laboratory parameters showing progressive biochemical improvement over time Serial laboratory parameters demonstrated progressive biochemical improvement over time in a patient with liver dysfunction. Notable reductions in bilirubin, transaminases, and GGT are observed, alongside stabilisation of INR and haemoglobin. Platelet count shows recovery from initial thrombocytopenia. ALP: alkaline phosphatase; ALT: alanine transaminase; ALT: alanine aminotransferase; GGT: gamma-glutamyl transferase; INR: international normalised ratio

TEST	15/8/25	29/8/25	24/9/25
Bilirubin (µmol/L)	170	377	47
ALP (U/L)	375	294	160
ALT (U/L)	182	88	60
AST (U/L)	270	180	130
GGT (U/L)	4464	1860	251
INR	1.19	1.10	0.99
HB (g/L)	129	129	129
Platelets (×10⁹/L)	93	447	348

**Figure 1:**
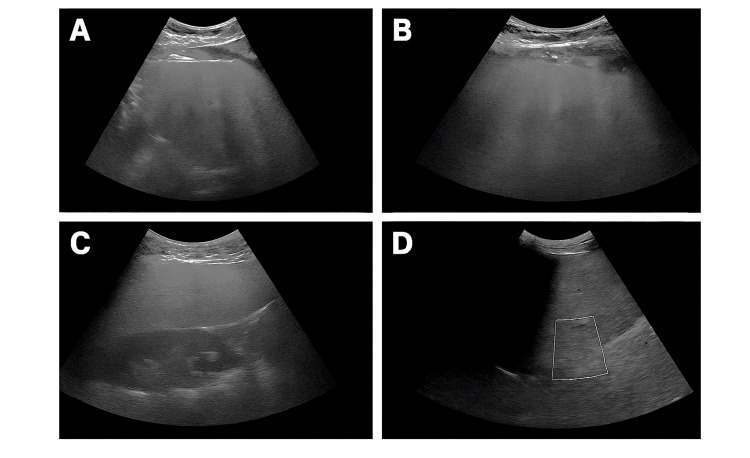
Ultrasound showing nodular liver surface with features suggestive of cirrhosis Ultrasound imaging of the liver demonstrating nodular surface architecture and heterogeneous echotexture, features suggestive of cirrhosis. Multiple grayscale images show surface contour irregularities, with panel D highlighting a region of interest.

Ultrasound imaging of the abdomen demonstrated a nodular liver surface, coarse echotexture, and mild splenomegaly, features suggestive of cirrhosis (Figure 1). No ascites or focal lesions were identified.

His Child-Pugh score at presentation was 7 (Child Class B), indicating a moderate degree of liver dysfunction and raising concern for possible early cirrhosis. According to National Institute for Health and Care Excellence (NICE) and British Liver Trust guidelines, the diagnostic approach to suspected cirrhosis involves a combination of liver biochemical testing, imaging modalities such as ultrasound or elastography, and validated scoring systems including Child-Pugh and MELD [6,8]. Although liver biopsy remains the gold standard for definitive diagnosis, it is generally reserved for cases with uncertain or conflicting clinical, laboratory, or imaging findings due to its invasive nature and associated risks.

Non-invasive diagnostic tools, including transient elastography (FibroScan), and serum fibrosis assessments, such as enhanced liver fibrosis (ELF) and Fibrosis-4 index (FIB-4), are increasingly recommended for the early detection of fibrosis and cirrhosis, including in individuals with alcohol use disorder [9,10]. Despite their value, these modalities are often underutilized in acute or emergency settings, where clinical priorities are dominated by the immediate management of alcohol withdrawal and medical stabilization. In this case, further confirmatory testing (such as FibroScan or liver biopsy) was not done acutely; however, given the correlation between the clinical presentation, biochemical abnormalities, and ultrasound features suggestive of cirrhosis, his fibrosis assessment as part of ongoing follow-up was scheduled as an outpatient. Recent NICE guidance encourages wider use of FibroScan outside specialist centers to enhance early detection and reduce diagnostic delays [11], and the patient remains under ongoing hepatology follow-up, where additional fibrosis assessment will be performed if clinically indicated.

A comprehensive etiological screen for chronic liver disease was performed. Viral serology (HBV, HCV), autoimmune markers (ANA, SMA, AMA), iron studies for hemochromatosis (ferritin, transferrin saturation), ceruloplasmin for Wilson’s disease, and alpha-1 antitrypsin levels were all within normal limits, effectively excluding metabolic, genetic, autoimmune, and viral causes of advanced liver disease. His BMI was within the normal range, and his HbA1c was normal, making nonalcoholic fatty liver disease (NAFLD)/Metabolic dysfunction-associated steatotic liver disease (MASLD) an unlikely contributor. These findings supported the impression that his liver injury was alcohol-related rather than secondary to an alternative metabolic or hereditary disorder.

Management

Management of ARLD involves three key stages: acute alcohol withdrawal treatment, psychosocial support, and hepatology follow-up.

Acute Withdrawal

The patient received a standard alcohol withdrawal regimen using chlordiazepoxide, guided by Clinical Institute Withdrawal Assessment of Alcohol, Revised (CIWA-Ar) scoring. Thiamine and other B vitamins were administered to prevent Wernicke encephalopathy. Electrolytes and hydration were closely monitored. This approach aligns with BMJ Best Practice and EASL guidelines, which recommend symptom-triggered benzodiazepine therapy and nutritional support during alcohol withdrawal [7,12].

*Psychosocial and Behavioural Support* 

Given the clear psychosocial trigger, bereavement counseling was initiated during admission. He was referred to community alcohol services and offered cognitive behavioral therapy (CBT). NICE guidelines emphasize integrated care involving addiction services, mental health support, and social interventions for patients with ARLD [6]. Multidisciplinary models addressing coexisting liver disease and alcohol use disorder have demonstrated improved outcomes [13].

Hepatology Follow-Up and Prognosis

An outpatient hepatology review was arranged to assess fibrosis progression, screen for complications (e.g., varices, hepatocellular carcinoma), and evaluate candidacy for long-term surveillance. Sustained abstinence was strongly encouraged, as studies show that even advanced liver injury may stabilize or partially regress with alcohol cessation, particularly in younger patients [14]. Integrated liver-alcohol clinics have emerged as promising models, combining hepatology, psychiatry, and addiction medicine, with early data suggesting improved engagement and reduced readmissions [15,16].

## Discussion

This case exemplifies the emerging trend of early-onset ARLD in young adults. While cirrhosis is traditionally linked to cumulative alcohol exposure, recent literature highlights the role of binge drinking, genetic predisposition, and psychosocial stressors in the progression of advanced liver disease, including features that may be suggestive of early cirrhosis in some individuals [2,17].

The patient’s rapid escalation in alcohol intake following bereavement underscores the need for holistic assessment and early intervention. Psychosocial factors, including trauma, mental health disorders, and socioeconomic stress, are increasingly recognized as contributors to alcohol misuse and harmful liver outcomes [18].

Compared to other published cases, this patient’s presentation was notable for the absence of decompensated features, such as ascites or encephalopathy, despite biochemical and radiological features suggestive of early cirrhosis. This highlights the importance of screening and early detection, particularly in patients presenting with alcohol withdrawal or psychiatric distress.

Non-invasive diagnostic tools, such as FibroScan and serum fibrosis panels, are recommended but remain underutilized in acute care settings [9,11]. A review by the British Society of Gastroenterology advocates routine screening for liver fibrosis in individuals with alcohol use disorder, even in the absence of symptoms [10].

Although the clinical picture strongly supported alcohol-related liver injury, alternative causes of advanced liver disease were considered. A comprehensive etiological evaluation excluded viral hepatitis, autoimmune liver disease, metabolic disorders, such as hemochromatosis and Wilson disease, and NAFLD/MASLD. Given his age, these differential diagnoses were important to assess before attributing findings solely to alcohol-related injury.

This case also raises important public health questions. Should young adults with high-risk drinking patterns be screened for liver disease? Can early intervention prevent progression to advanced fibrosis or cirrhosis? As ARLD becomes more prevalent among younger populations, proactive strategies, including public health education, integrated care models, and early hepatology referral, are needed to reduce the burden [19,20].

## Conclusions

This case highlights the possibility of significant alcohol-related liver injury in a young adult after a relatively short period of high-risk drinking. The clinical, laboratory, and imaging features raised concern for early cirrhosis, underscoring the importance of prompt assessment and coordinated multidisciplinary care. Psychosocial support and structured hepatology follow-up were key components of management, emphasizing the value of addressing both medical and behavioral contributors to alcohol use.

Clinicians should maintain a high index of suspicion for alcohol-related liver disease in younger patients presenting with alcohol withdrawal, particularly when recent psychosocial stressors are identified. From a public health perspective, early engagement, education, and integrated care models may help reduce the growing burden of alcohol-related liver disease among younger populations. Ongoing research is needed to better characterize predictors of rapid progression and to inform strategies for early detection and intervention.

## References

[1] Williams R, Ashton K, Aspinall R (2020). Addressing liver disease in the UK: a blueprint for attaining excellence in health care and reducing premature mortality from lifestyle issues of excess consumption of alcohol, obesity, and viral hepatitis. Lancet Gastroenterol Hepatol.

[2] Rehm J, Mathers C, Popova S, Thavorncharoensap M, Teerawattananon Y, Patra J (2009). Global burden of disease and injury and economic cost attributable to alcohol use and alcohol-use disorders. Lancet.

[3] Monnig MA, Treloar Padovano H, Monti PM (2024). Alcohol-associated liver disease and behavioral and medical cofactors: unmet needs and opportunities. Front Public Health.

[4] Kang J, Park SH, Khanam M, Park SB, Shin S, Seo W (2025). Impact of binge drinking on alcoholic liver disease. Arch Pharm Res.

[5] (2025). British Liver Trust. Cirrhosis treatments and complications. https://britishlivertrust.org.uk/information-and-support/liver-conditions/cirrhosis/treatments-and-complications/.

[6] (2025). National Institute for Health and Care Excellence (NICE). Alcohol-use disorders: diagnosis and management of physical complications. Clinical guideline. Reference number: CG100. Clinical guideline CG100 [Internet.

[7] European Association for the Study of the Liver (2018). EASL Clinical Practice Guidelines: management of alcohol-related liver disease. J Hepatol.

[8] Mirijello A, D'Angelo C, Ferrulli A (2015). Identification and management of alcohol withdrawal syndrome. Drugs.

[9] Shah ND, Ventura-Cots M, Abraldes JG (2019). Alcohol-related liver disease is rarely detected at early stages compared with liver diseases of other etiologies worldwide. Clin Gastroenterol Hepatol.

[10] Parker R, Allison M, Anderson S (2023). Quality standards for the management of alcohol-related liver disease: consensus recommendations from the British Association for the Study of the Liver and British Society of Gastroenterology ARLD special interest group. BMJ Open Gastroenterol.

[11] (2025). National Institute for Health and Care Excellence (NICE). Cirrhosis: assessment and management. NICE guideline. Reference number: NG50. Clinical guideline.

[12] BMJ Best Practice (2025). BMJ Best Practice. Alcohol withdrawal. https://bestpractice.bmj.com/topics/en-gb/549.

[13] Herms Q, Gratacós-Ginès J, Pose E (2025). Alcohol-related liver disease. Gastroenterol Rep (Oxf).

[14] Altamirano J, López-Pelayo H, Michelena J (2017). Alcohol abstinence in patients surviving an episode of alcoholic hepatitis: prediction and impact on long-term survival. Hepatology.

[15] Abusuliman M, Milgrom Y, Mellinger J, Parker R (2025). Management of alcohol use disorder in alcohol-related liver disease. Frontline Gastroenterology.

[16] Winder GS, Fernandez AC, Mellinger JL (2022). Integrated care of alcohol-related liver disease. J Clin Exp Hepatol.

[17] Tapper EB, Parikh ND (2018). Mortality due to cirrhosis and liver cancer in the United States, 1999-2016: observational study. BMJ.

[18] Danpanichkul P, Pang Y, Diaz LA (2025). Young adults and alcohol-associated liver cancer: incidence and death from 2000 to 2021. Cancers (Basel).

[19] Parker R, Arab JP, Lazarus JV, Bataller R, Singal AK (2025). Public health policies to prevent alcohol-related liver disease. Nat Rev Gastroenterol Hepatol.

[20] Younossi ZM, Zelber-Sagi S, Henry L, Gerber LH (2023). Lifestyle interventions in nonalcoholic fatty liver disease. Nat Rev Gastroenterol Hepatol.

